# Impairment of spatial memory accuracy improved by *Cbr1* copy number resumption and GABA_B_ receptor-dependent enhancement of synaptic inhibition in Down syndrome model mice

**DOI:** 10.1038/s41598-020-71085-9

**Published:** 2020-08-25

**Authors:** Fumiko Arima-Yoshida, Matthieu Raveau, Atsushi Shimohata, Kenji Amano, Akihiro Fukushima, Masashi Watanave, Shizuka Kobayashi, Satoko Hattori, Masaya Usui, Haruhiko Sago, Nobuko Mataga, Tsuyoshi Miyakawa, Kazuhiro Yamakawa, Toshiya Manabe

**Affiliations:** 1 Division of Neuronal Network, Institute of Medical Science, University of Tokyo, Tokyo, 108-8639 Japan; 2 Laboratory for Neurogenetics, RIKEN Center for Brain Science, Wako, Saitama 351-0198 Japan; 3 Division of Systems Medical Science, Institute for Comprehensive Medical Science, Fujita Health University, Toyoake, Aichi 470-1192 Japan; 4 Research Resources Division, RIKEN Center for Brain Science, Wako, Saitama 351-0198 Japan; 5 Center for Maternal-Fetal, Neonatal and Reproductive Medicine, National Center for Child Health and Development, Tokyo, 157-8535 Japan; 6 Department of Neurodevelopmental Disorder Genetics, Institute of Brain Sciences, Nagoya City University Graduate School of Medical Sciences, Nagoya, Aichi 467-8601 Japan

**Keywords:** Diseases of the nervous system, Learning and memory, Molecular neuroscience, Neuronal physiology, Synaptic plasticity, Synaptic transmission, Neurological disorders, Neurological disorders, Neuroscience, Diseases, Neurology, Genetics, Behavioural genetics, Neurodevelopmental disorders

## Abstract

Down syndrome is a complex genetic disorder caused by the presence of three copies of the chromosome 21 in humans. The most common models, carrying extra-copies of overlapping fragments of mouse chromosome 16 that is syntenic to human chromosome 21, are Ts2Cje, Ts1Cje and Ts1Rhr mice. In electrophysiological analyses using hippocampal slices, we found that the later phase of the depolarization during tetanic stimulation, which was regulated by GABA_B_ receptors, was significantly smaller in Ts1Cje and Ts2Cje mice than that in WT controls but not in Ts1Rhr mice. Furthermore, isolated GABA_B_ receptor-mediated inhibitory synaptic responses were larger in Ts1Cje mice. To our knowledge, this is the first report that directly shows the enhancement of GABA_B_ receptor-mediated synaptic currents in Ts1Cje mice. These results suggest that GABA_B_ receptor-mediated synaptic inhibition was enhanced in Ts1Cje and Ts2Cje mice but not in Ts1Rhr mice. The *Cbr1* gene, which is present in three copies in Ts1Cje and Ts2Cje but not in Ts1Rhr, encodes carbonyl reductase that may facilitate GABA_B_-receptor activity through a reduction of prostaglandin E2 (PGE2). Interestingly, we found that a reduction of PGE2 and an memory impairment in Ts1Cje mice were alleviated when only *Cbr1* was set back to two copies (Ts1Cje;*Cbr1*^+/+/−^). However, the GABA_B_ receptor-dependent enhancement of synaptic inhibition in Ts1Cje was unaltered in Ts1Cje;*Cbr1*^+/+/−^ mice. These results indicate that *Cbr1* is one of the genes responsible for DS cognitive impairments and the gene(s) other than *Cbr1*, which is included in Ts1Cje but not in Ts1Rhr, is responsible for the GABA_B_ receptor-dependent over-inhibition.

## Introduction

Down syndrome (DS) is a complex genetic disorder caused by the presence of three copies of the whole or part of the chromosome 21 in humans. The resulting gene-dosage imbalance is responsible for a variety of phenotypes affecting various organs and notably leads to intellectual disability in the whole DS population^1,2^. Among others, hippocampus-dependent learning and memory deficits affect verbal and non-verbal cognition as well as spatial memory skills^3,4^. Deciphering the pathways and mechanisms at play in these dysfunctions in humans, however, remains a challenge.

Human chromosome 21 possesses regions of synteny in the mouse with a high conservation of genes in similar order and relative orientation on chromosomes 10, 16 and 17 (MMU10, MMU16 and MMU17). The largest region of synteny is found on MMU16 and as such has been the main target for the generation of model mice. The most common models, carrying extra-copies of overlapping fragments of MMU16, are (from the largest to shortest): Ts65Dn, Ts2Cje, Ts1Cje and Ts1Rhr (Supplementary Fig. 1)^5–7^. Although Ts65Dn carries the distal end of MMU16 as a small marker chromosome conforming a partial trisomy and shows male infertility and poor transmission of the marker chromosome, Ts2Cje carries an equivalent trisomic region of Ts65Dn but it has been translocated to MMU12 forming a Robertsonian chromosome and this stable rearrangement confers fertility in males and increases the frequency of transmitted segmental trisomy through the female germline^8^. These models have been used in comparative studies in order to understand the regions of interest for the various DS-like phenotypes, with the largest duplication leading to the most severe impairments overall^9,10^. For instance, Ts1Cje mice display craniofacial malformation and learning and memory deficits mimicking DS^6,11,12^. Even though the mechanistic origins of these phenotypes remain unclear, the Ts1Cje model helped identify cellular and molecular abnormalities likely involved in DS: aberrant changes in neuronal dendritic spines, synaptic plasticity and neurogenesis^12–14^. In contrast, the Ts1Rhr model carrying a shorter triplicated region shows milder phenotypes^12,15,16^. Comparative studies with these models and subtractive approaches using knockout (KO) lines to resume gene copy number in DS model mice allow the identification of candidate genes^17^.

Although the origin of brain dysfunction in DS is not understood very well to date, several studies have pointed out that an imbalance between excitation and inhibition could be a dominant player. For instance, deficits of synaptic plasticity and memory can be improved by treatments using γ-aminobutyric acid type A (GABA_A_)^18,19^- as well as γ-aminobutyric acid type B (GABA_B_)^20^-receptor antagonists. A few other drugs have so far been tested in rodent models and humans, among which epigallocatechin gallate has shown interesting improvements especially when combined with environmental enrichment^21^. Even though the effect of epigallocatechin gallate has been assumed to be reached through the kinase DYRK1A^22^, it also has the ability to inhibit another candidate molecule for DS: the carbonyl reductase Cbr1^23^. *Cbr1* is located on MMU16 and is present in three copies in Ts1Cje but not in Ts1Rhr mice. As such, it is an interesting candidate for phenotypes specific to the Ts1Cje model. Here, we investigated whether GABA_A_ and/or GABA_B_ receptor-mediated over-inhibition could be observed in Ts1Cje and Ts1Rhr mice and whether Cbr1 was a key player in these changes. For that purpose, we designed a combination of electrophysiology and behavioral phenotyping strategy and used a genetic resumption approach to set *Cbr1* back to two copies in DS model mice.

## Results

### Depolarization during high-frequency stimulation is reduced in Ts1Cje and Ts2Cje but not in Ts1Rhr mice

To identify regions responsible for memory impairments in the trisomic segments of Ts1Cje, Ts2Cje and Ts1Rhr mouse models (Supplementary Fig. 1), synaptic plasticity was compared between the genotypes (Supplementary Fig. 2). Long-term potentiation (LTP) induced by tetanic stimulation (100 Hz, 1 s) in the CA1 region of hippocampal slices was mostly intact in all Ts1Cje (Supplementary Fig. 2a), Ts2Cje (Supplementary Fig. 2b) and Ts1Rhr (Supplementary Fig. 2c) mice, suggesting that the trisomic manipulation in these models did not impair long-term synaptic plasticity significantly.

**Figure 1:**
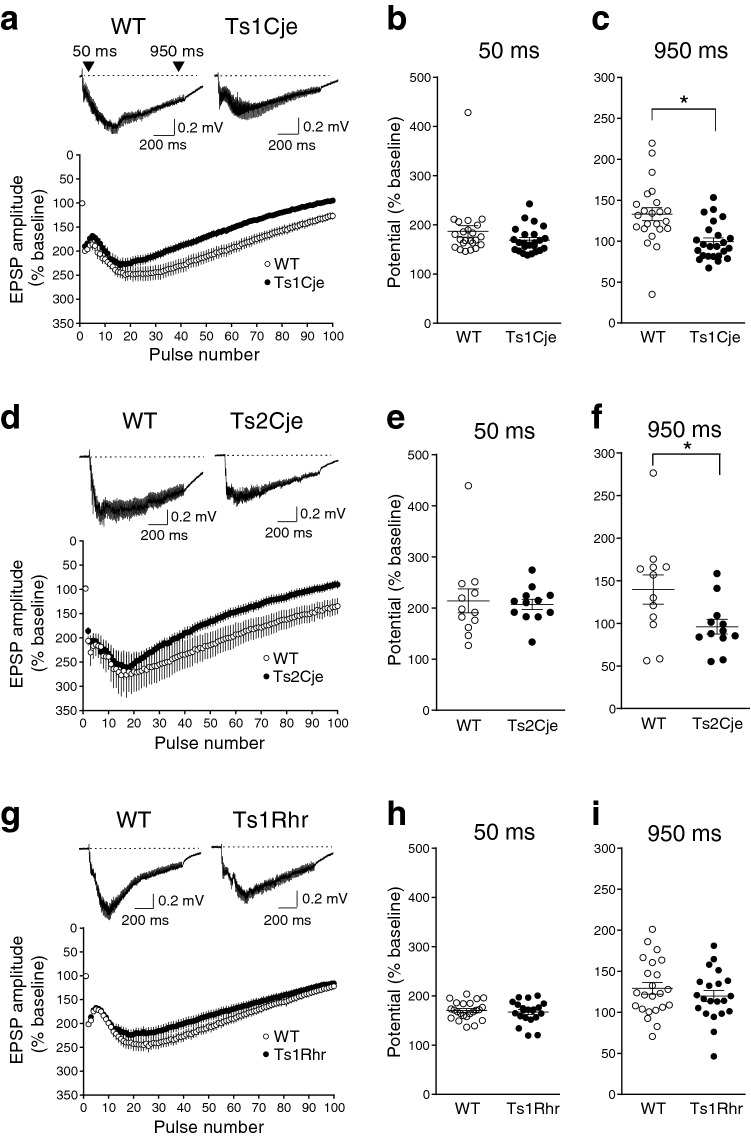
The later phase of the depolarization envelope was decreased in Ts1Cje and Ts2Cje, but not in Ts1Rhr mice. (**a**,**d**,**g**) Sample traces of EPSPs during tetanic stimulation (upper) and averaged traces (lower) in Ts1Cje (**a**: closed circles), Ts2Cje (**d**: closed circles) and Ts1Rhr (**g**: closed circles) mice as well as WT mice (**a**,**d**,**g**: open circles). The recorded potentials were plotted as a function of pulse number during tetanic stimulation (100 Hz, 1 s) in the lower panels. (**b**,**c**,**e**,**f**,**h**,**i**) Depolarization measured at 50 ms and 950 ms after the first pulse of tetanic stimulation was plotted for each experiment. No statistical difference was detected in the depolarization envelope at 50 ms (earlier phase) in any genotypes (**b**,**e**,**h**), whereas the depolarization envelope at 950 ms (later phase) was significantly reduced in Ts1Cje (**c**) and Ts2Cje (**f**) mice, but not in Ts1Rhr mice (**i**). Statistical significance was assessed by Student’s t-test.

In contrast, depolarization during high-frequency stimulation (depolarization envelope) 950 ms after the first pulse of tetanic stimulation (Fig. 1) was significantly smaller in Ts1Cje [Fig. 1a,c (*n* = 24)] and Ts2Cje [Fig. 1d,f (*n* = 12)] mice than in their wild-type littermate controls (WT mice) [Fig. 1a,c (*n* = 23), d,f (*n* = 12)], while there was no difference between Ts1Rhr (*n* = 21) and WT (*n* = 23) mice (Fig. 1g,i). These results indicate that the responsible gene(s) for the reduced depolarization envelope was localized in the trisomic region common to Ts1Cje and Ts2Cje mice but not in that of Ts1Rhr mice (see Supplementary Fig. 1). On the other hand, the earlier phase of the depolarization envelope (50 ms after the first pulse of tetanic stimulation) was not statistically different between WT and Ts1Cje (Fig. 1b); WT and Ts2Cje (Fig. 1e); and WT and Ts1Rhr (Fig. 1h) mice.

### The later phase of the depolarization envelope is regulated by GABA_B_ receptors

**Figure 2:**
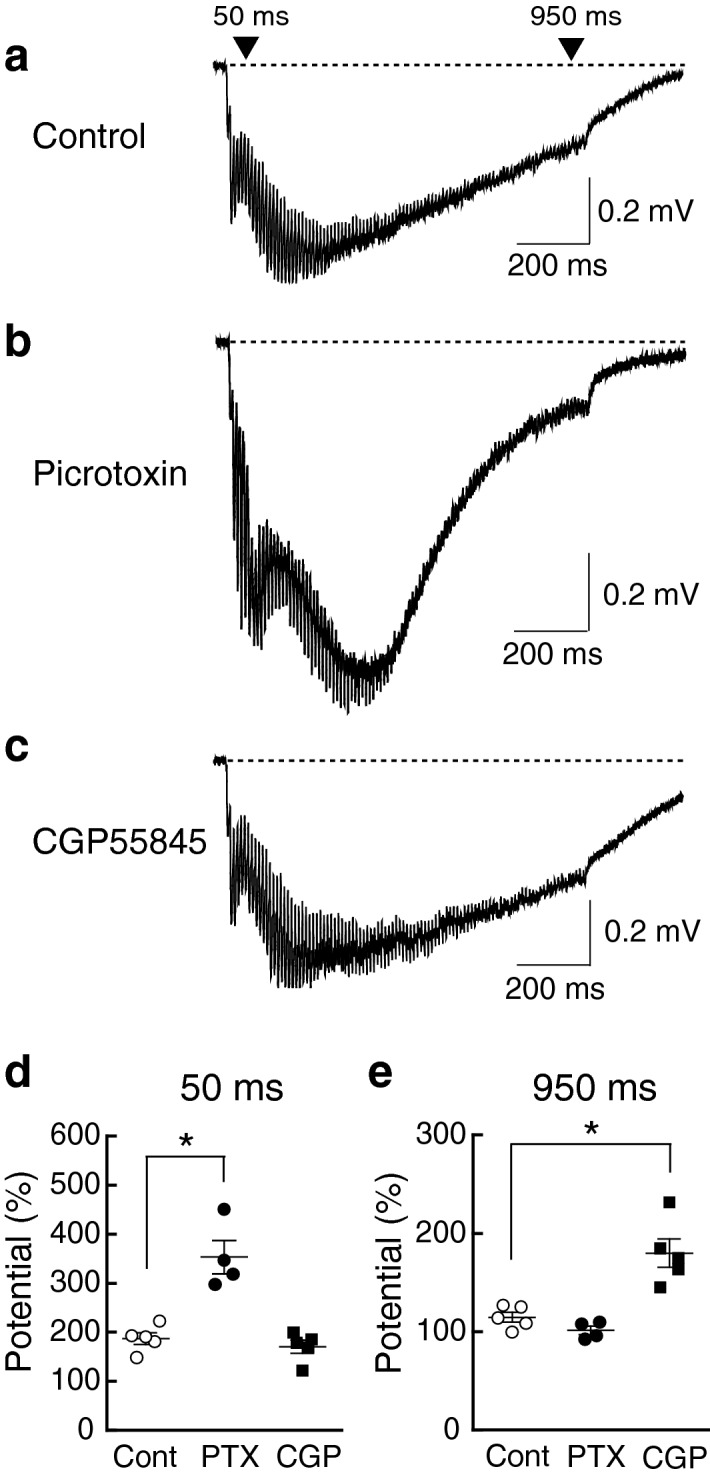
The earlier phase of the depolarization envelope was regulated by GABA_A_ receptors, whereas the later phase was regulated by GABA_B_ receptors. (**a**–**c)** Sample traces of depolarization envelopes. In the presence of the GABA_A_-receptor antagonist picrotoxin (**b**), the earlier phase became larger compared to that in the absence of picrotoxin (**a**). In the presence of the GABA_B_-receptor antagonist CGP55845 (**c**), the later phase became larger compared to that in the absence of CGP55845 (**a**). (**d**,**e**) Depolarization measured at 50 ms (**d**) and 950 ms (**e**) after the first pulse of tetanic stimulation in the absence (Cont) and the presence of picrotoxin (PTX) or CGP55845 (CGP) was plotted for each experiment. Statistical significance was assessed by one-way ANOVA followed by Bonferroni post-hoc test.

In order to clarify the receptor mechanism of the reduced depolarization envelope, we performed pharmacological experiments (Fig. 2). We focused on GABAergic synaptic transmission because previous studies reported the involvement of GABA_A_^18,19^ and GABA_B_^20^ receptors in the phenotypes of DS. Using C57BL/6 J mice, we examined the effect of 100 µM picrotoxin, a GABA_A_-receptor antagonist, on depolarization envelopes (Fig. 2a,b) and found that the earlier phase of the depolarization (50 ms) was increased [Fig. 2d: PTX (*n* = 4)] but the later phase (950 ms) was unchanged [Fig. 2e: PTX (*n* = 4)] compared to control (Cont) (*n* = 5). Next, we examined the effect of 2 µM CGP55845, a GABA_B_-receptor antagonist, on the depolarization envelope (Fig. 2a, c) and found that the earlier phase was unchanged [Fig. 2d: CGP (*n* = 5)] but the later phase was increased [Fig. 2e: CGP (*n* = 5)] compared to control (Cont) (*n* = 5). These results suggest that GABA_B_ receptor-mediated synaptic inhibition may be enhanced in Ts1Cje and Ts2Cje mice. In the following experiments, we examined only Ts1Cje mice, since their trisomic region was shorter than that of Ts2Cje mice but the electrophysiological phenotype was indistinguishable between Ts1Cje and Ts2Cje mice.

### The difference in the depolarization envelope in Ts1Cje mice is canceled by a GABA_B_-receptor antagonist

**Figure 3:**
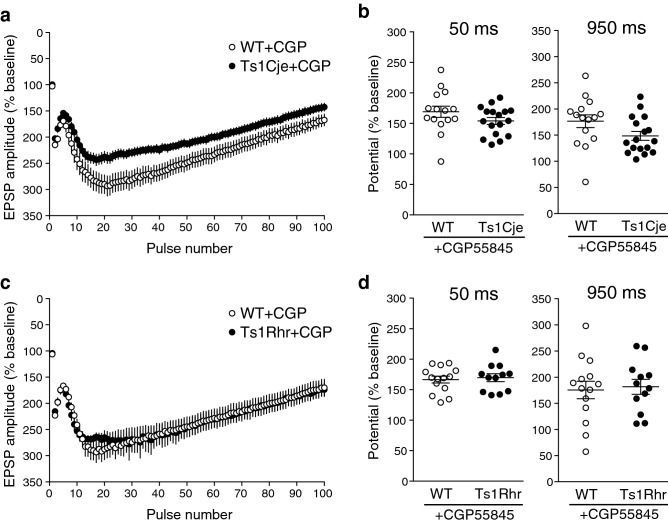
The effect of the GABA_B_-receptor antagonist on the depolarization envelope in WT, Ts1Cje and Ts1Rhr mice. (**a**) Averaged traces of depolarization envelopes of WT (open circles) and Ts1Cje (closed circles) mice in the presence of the GABA_B_-receptor antagonist CGP55845. (**b**) In the presence of CGP55845, there was no significant difference in the potential at either 50 ms or 950 ms in Ts1Cje mice. (**c**) Averaged traces of depolarization envelopes of WT (open circles) and Ts1Rhr (closed circles) mice in the presence of CGP55845. (**d**) In the presence of CGP55845, there was no significant difference in the potential at either 50 ms or 950 ms in Ts1Rhr mice. Statistical significance was assessed by Student’s t-test.

We next examined the effect of CGP55845 on the depolarization envelope in Ts1Cje and Ts1Rhr mice. In control experiments, as shown above (Fig. 1), the later phase of the depolarization envelope was significantly smaller in Ts1Cje mice than that in WT mice (Fig. 1a). In the presence of the GABA_B_-receptor antagonist, the later phase of the depolarization envelope in WT (*n* = 15) and Ts1Cje (*n* = 17) mice became larger (Fig. 3a, compare to Fig. 1a), but the change was more remarkable in Ts1Cje mice and the difference observed in control conditions disappeared (Fig. 3b: 950 ms). There was no difference at the earlier phase of the depolarization envelope (Fig. 3b: 50 ms) as in the absence of CGP55845. In contrast, the later phase of the depolarization envelope in WT (*n* = 14) and Ts1Rhr (*n* = 12) mice became larger in the presence of the antagonist to a similar extent (Fig. 3c), and there was still no significant difference between them (Fig. 3d: 950 ms). There was no difference at the earlier phase of the depolarization envelope (Fig. 3d: 50 ms) as in the absence of CGP55845. This result strongly suggests that GABA_B_ receptor-mediated inhibition is stronger in Ts1Cje mice compared to WT and Ts1Rhr mice. The difference observed between WT and Ts1Cje mice in the presence of CGP55845 (Fig. 3a, around 20–50 pulses) might be caused by the residual difference in the GABA_A_ receptor-mediated synaptic inhibition that was not sensitive to CGP55845.

### GABA_B_ receptor-mediated synaptic inhibition is enhanced in Ts1Cje mice

**Figure 4:**
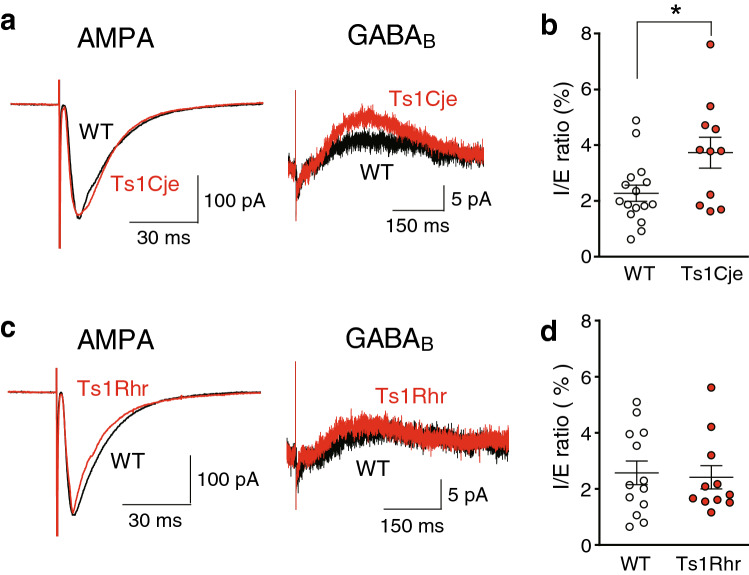
GABA_B_ receptor-mediated inhibition was stronger in Ts1Cje mice than that in WT and Ts1Rhr mice. (**a**) Sample traces of AMPA receptor-mediated EPSCs (left) and GABA_B_ receptor-mediated IPSCs (right) in WT (black) and Ts1Cje (red) mice. (**b**) The I/E ratio was larger in Ts1Cje mice than that in WT mice. (**c**) Sample traces of AMPA receptor-mediated EPSCs (left) and GABA_B_ receptor-mediated IPSCs (right) in WT (black) and Ts1Rhr (red) mice. (**d**) The I/E ratio was comparable between WT and Ts1Rhr mice. The value of the I/E ratio was first corrected by Smirnov-Grubbs test and then compared between the genotypes by Student’s t-test.

To compare GABA_B_ receptor-mediated inhibitory synaptic responses directly, inhibitory postsynaptic currents (IPSCs) were recorded from CA1 pyramidal cells with the whole-cell patch-clamp recording technique using WT, Ts1Cje and Ts1Rhr mice (Fig. 4). To normalize the stimulus strength, we first recorded α-amino-3-hydroxy-5-methyl-4-isoxazolepropionic acid (AMPA) receptor-mediated excitatory postsynaptic currents (EPSCs) at − 60 mV with comparable amplitudes between the genotypes (Fig. 4a,c: AMPA). Picrotoxin was present throughout the experiment to block GABA_A_ receptor-mediated responses. We then recorded GABA_B_ receptor-mediated IPSCs by pharmacologically blocking excitatory synaptic responses by 6-cyano-7-nitroquinoxaline-2,3-dione (CNQX), an AMPA-receptor antagonist, and d-(−)-2-amino-5-phosphonopentanoic acid (D-AP5), an NMDA-receptor antagonist, without changing the stimulus strength in Ts1Cje (Fig. 4a: GABA_B_) and Ts1Rhr (Fig. 4c: GABA_B_) mice. The ratio of GABA_B_ receptor-mediated IPSCs to AMPA receptor-mediated EPSCs (I/E ratio) was significantly larger in Ts1Cje mice (*n* = 11) than that in WT mice (*n* = 16) (Fig. 4b), whereas the ratio in Ts1Rhr mice (*n* = 11) was comparable to that in WT mice (*n* = 13) (Fig. 4d). AMPA receptor-mediated synaptic responses were indistinguishable between the genotypes, because the stimulus strength required to evoke similar EPSP slopes in the field-potential recording was not statistically different (WT vs. Ts1Cje mice: 1.57 ± 0.11 V (n = 23) vs. 1.53 ± 0.06 V (n = 25), respectively, *p* = 0.773; WT vs. Ts1Rhr: 1.61 ± 0.11 V (n = 23) vs. 1.67 ± 0.12 V (n = 20), respectively, *p* = 0.718, Student’s t-test). Thus, these results indicate that GABA_B_ receptor-mediated inhibition was enhanced in Ts1Cje mice but not in Ts1Rhr mice, and that responsible gene(s) for these phenotypes is located in the trisomic region of Ts1Cje but not in that of Ts1Rhr.

### Hyperactivity in Ts1Cje mice

**Figure 5:**
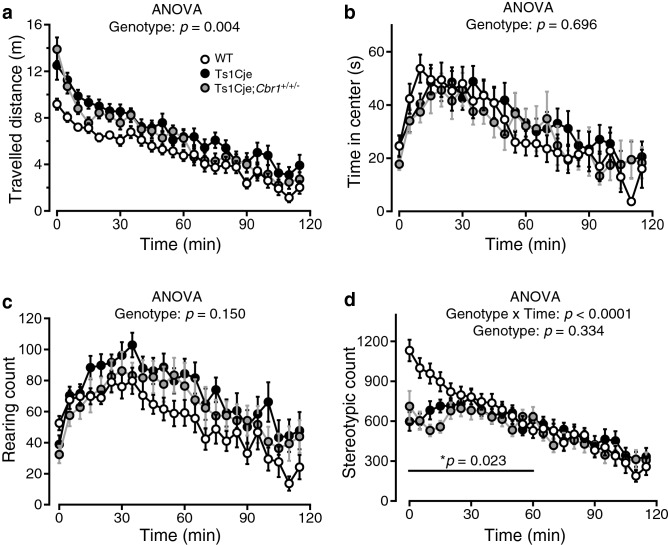
Hyper-locomotion and decreased stereotypic events in the open-field test were not affected by *Cbr1* resumption to two copies. (**a**) In the open-field task, the distance travelled by Ts1Cje mice was significantly longer than that by their WT littermates. The distance travelled by Ts1Cje;*Cbr1*^+/+/−^ mice was similarly increased and did not differ significantly from Ts1Cje mice. (**b**, **c**) The time spent in the center area (**b**) and the number of rearing events (**c**) were not significantly different between the three groups. (**d**) The number of stereotypic events was significantly lower in Ts1Cje mice than that in WT littermates, especially during the first 60 min of the test. The number of stereotypic events of Ts1Cje;*Cbr1*^+/+/−^ mice was significant decreased, which was not significantly different from that of Ts1Cje littermates. Statistical significance was assessed using two-way repeated measures ANOVA.

In our previous work, an extensive behavioral characterization of Ts1Cje mice revealed a significant increase in locomotor activity when they were exposed to an unfamiliar environment^24^. In the present study, we confirmed it showing that Ts1Cje mice travelled significantly longer distances than WT mice (Fig. 5a). The time spent in the center of the open field (Fig. 5b) and the number of rearing (Fig. 5c) tended to be slightly larger in Ts1Cje mice, although the difference did not reach the significance level. The number of stereotypic events, which were repetitive movements detected by laser sensors, was significantly decreased in Ts1Cje mice, and this effect was particularly remarkable during the first 60 min of exploration (Fig. 5d).

### *Cbr1 *as a candidate gene for DS and copy number resumption of *Cbr1* in Ts1Cje mice

*Cbr1* is localized in the trisomic region of Ts1Cje but outside of that of Ts1Rhr (Supplementary Fig. 1) and is a good candidate gene for DS. To investigate the involvement of *Cbr1* in the phenotypes observed in Ts1Cje mice, we designed a genetic approach of the gene copy number resumption by crossing the Ts1Cje model with a *Cbr1* KO line, as we did for another candidate gene in a previous study^17^. For that purpose, we generated a *Cbr1* KO line using in-vivo Cre/loxP recombination (Supplementary Fig. 3a) and crossed this KO mouse with the Ts1Cje line to generate compound Ts1Cje;*Cbr1*^+/+/−^ in which only *Cbr1* is set back to two copies (Supplementary Fig. 3b). Gene-expression analysis of *Cbr1* confirmed a significant increase of *Cbr1* RNA levels in brain extracts of Ts1Cje mice (*n* = 3) and a significant decrease in *Cbr1*^+/−^ samples (*n* = 3), whereas the *Cbr1* mRNA level in Ts1Cje;*Cbr1*^+/+/−^ brains (*n* = 6) was comparable to that of WT littermate brains (*n* = 3) (Supplementary Fig. 3c, d), hence confirming the successful recovery in the compound mice.

Ahead of the behavior screening, we investigated the general health and neuromuscular condition in these mice at the adult stage (21 weeks old; *N* = 20 for each genotype in all experiments in this paragraph). The body weight of Ts1Cje (32.2 ± 0.5 g) and Ts1Cje;*Cbr1*^+/+/−^ (32.2 ± 0.7 g) mice was significantly lower (*p* < 0.001, one-way ANOVA) than that of their WT littermates (36.5 ± 0.7 g). We did not observe significant differences (*p* = 0.956, one-way ANOVA) in body temperature between WT (35.5 ± 0.2 °C), Ts1Cje (35.5 ± 0.2 °C) and Ts1Cje;*Cbr1*^+/+/−^ (35.5 ± 0.2 °C) mice. There was a mild yet not significant difference in muscular strength between the groups in the wire-hang test (latency to fall: WT 31.1 ± 5.0 s; Ts1Cje 28.9 ± 4.2 s; Ts1Cje;*Cbr1*^+/+/−^ 19.9 ± 3.1 s; *p* = 0.139, one-way ANOVA), and the grip-strength test (WT 0.913 ± 0.034 N; Ts1Cje 0.978 ± 0.040 N; Ts1Cje;*Cbr1*^+/+/−^ 0.920 ± 0.041 N) showed no significant differences (*p* = 0.434, one-way ANOVA), indicating that neuromuscular function was conserved in Ts1Cje mice and not affected by *Cbr1* copy number variation.

### *Cbr1* resumption to two copies rescues cognitive deficiency but not hyperactivity in Ts1Cje mice

The resumption to two copies of *Cbr1* did not alleviate hyperactivity in Ts1Cje mice (*N* = 20) because Ts1Cje;*Cbr1*^+/+/−^ mice (*N* = 20) also travelled significantly longer distances (Fig. 5a) and showed significantly less stereotypic events (Fig. 5d) than WT littermates (*N* = 20). For these parameters, Ts1Cje and Ts1Cje;*Cbr1*^+/+/−^ did not differ significantly, indicating that these phenotypes are not likely to be caused by a triplication of *Cbr1*. Furthermore, the time spent in the center of the open field (Fig. 5b) and the number of rearing (Fig. 5c) were indistinguishable between WT, Ts1Cje and Ts1Cje;*Cbr1*^+/+/−^ mice.

**Figure 6:**
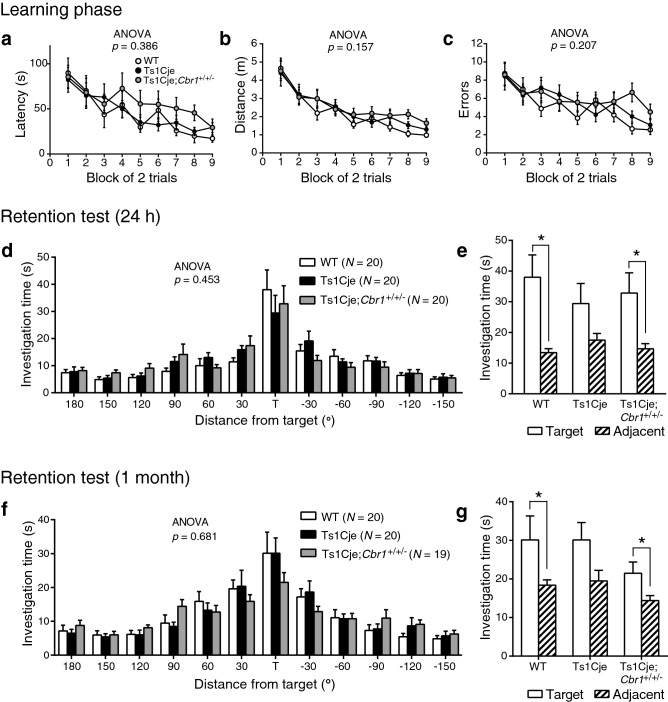
Deficit in spatial memory accuracy of Ts1Cje mice in the Barnes-maze task was improved partially by *Cbr1* resumption to two copies. (**a**–**c**) In the learning phase of the Barnes-maze task, the latency (**a**), distance (**b**) and number of errors (**c**) to reach the target did not differ significantly between Ts1Cje, Ts1Cje;*Cbr1*^+/+/−^ and WT mice. (**d**) In the probe test 1 day after the last training session, the time spent searching for the target and non-target holes was not significantly different between the three groups. (**e**) Whereas WT and Ts1Cje;*Cbr1*^+/+/−^ mice were able to discriminate between the target hole versus adjacent ones (non-target at ± 30°), this discrimination index did not reach the significance level in Ts1Cje mice. (**f**) In the probe test 1 month later, the time spent searching for the different holes of the maze was not significantly different between the three groups. (**g**) WT and Ts1Cje;*Cbr1*^+/+/−^ mice were able to discriminate the target hole from the adjacent ones whereas Ts1Cje mice failed to show a significant preference for the target hole. Statistical significance was assessed using two-way repeated measures ANOVA (**a**–**d**,**f**) or paired t-test (**e**,**g**).

Previous reports also identified learning and memory deficits in Ts1Cje mice^6,12^. We thus investigated whether the copy number resumption of *Cbr1* would affect these behavioral phenotypes in Ts1Cje mice. In order to investigate the hippocampus-dependent performance in a spatial learning and memory task, we used the Barnes-maze task^25^. In the learning phase, where animals were challenged to learn the position of an escape box, the latency, travelled distance and number of errors to reach the target hole decreased progressively with the consecutive trials (two-way repeated measures ANOVA, “time” effect *p* < 0.001) (Fig. 6a–c). We did not observe significant differences between Ts1Cje (*N* = 20), Ts1Cje;*Cbr1*^+/+/−^ (*N* = 20) and WT mice (*N* = 20), suggesting that the learning ability in these model mice was conserved. In the probe test 1 day after the final training session, the exploration across the different holes showed a similar pattern in the different groups of genotypes (Fig. 6d). The latency, distance and number of errors to reach the target for the first time were mildly yet not significantly larger in Ts1Cje and Ts1Cje;*Cbr1*^+/+/−^ (*p* = 0.123, *p* = 0.103, *p* = 0.214, respectively). However, Ts1Cje;*Cbr1*^+/+/−^ mice were able to significantly discriminate the target hole from the two adjacent ones as WT mice, while the significant difference in this behavior was not observed in Ts1Cje mice (Fig. 6e). On a month later, we challenged the long-term retention by another probe-test session. In a similar way to the first probe test performed 1 day after the final training session, the pattern of exploration across the board did not differ significantly between WT (*N* = 20), Ts1Cje (*N* = 20) and Ts1Cje;*Cbr1*^+/+/−^ (*N* = 19) mice (Fig. 6f). The latency, distance and number of errors to reach the target for the first time were mildly yet not significantly larger in Ts1Cje and Ts1Cje;*Cbr1*^+/+/−^ (*p* = 0.410, *p* = 0.121, *p* = 0.206, respectively). Comparing the amount of time spent on the target and adjacent holes, however, revealed that, unlike WT and Ts1Cje;*Cbr1*^+/+/−^ mice, Ts1Cje mice did not significantly discriminate the target hole from the two adjacent ones (Fig. 6g). Taken together, these results indicate a deficit in spatial memory accuracy in Ts1Cje mice and suggest that the resumption of *Cbr1* to two copies is able to recover this phenotype.

### Prostaglandin E2 and F2a are decreased in the hippocampus of Ts1Cje mice and *Cbr1* resumption partially improves this phenotype

**Figure 7:**
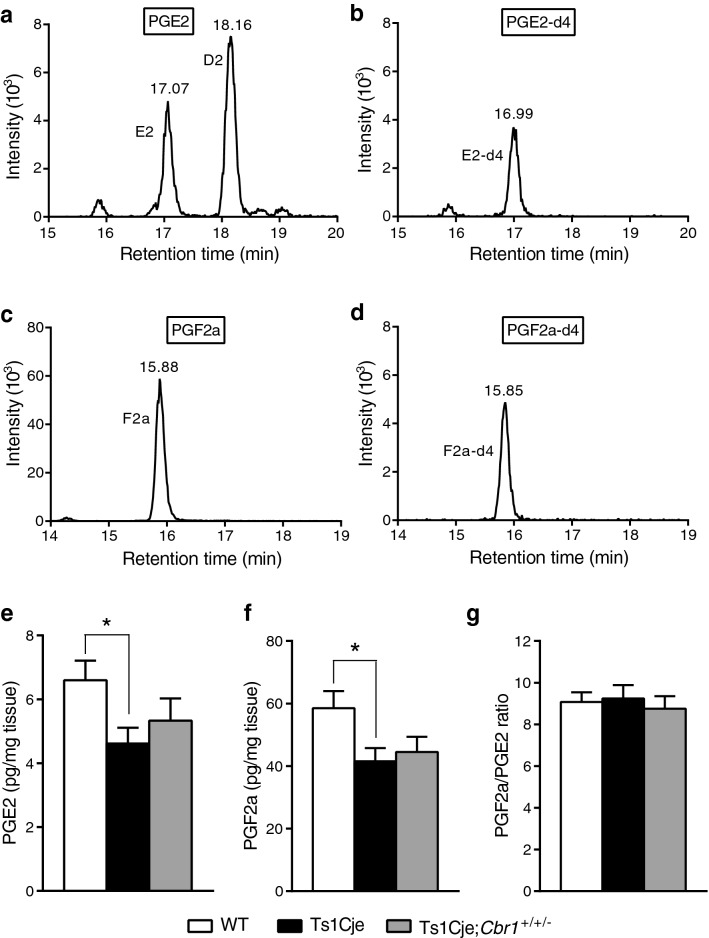
Prostaglandin E2 and F2a were significantly decreased in the hippocampus of Ts1Cje mice, which was not rescued in Ts1Cje;*Cbr1*^+/+/−^ mice. (**a**,**b**) Representative chromatograms show traces of endogenous prostaglandin E2 (PGE2) (**a**) and internal standard PGE2-d4 (**b**) used for precise quantification of PGE2 in mouse hippocampus extracts. (**c**,**d**) Representative chromatograms show traces of endogenous PGF2a (**c**) and internal standard PGF2a-d4 (**d**) used for precise quantification of PGF2a in mouse hippocampus extracts. **e** Quantitative analysis showed a significant decrease in PGE2 in the hippocampus of Ts1Cje mice (*n* = 12). The PGE2 level was also decreased in Ts1Cje;*Cbr1*^+/+/−^ samples (*n* = 14), but the difference did not reach the statistical significance compared to either WT (*n* = 14) or Ts1Cje littermates. **f** PGF2a was significantly reduced in the hippocampus of Ts1Cje mice and tended to be decreased to a comparable extent in Ts1Cje;*Cbr1*^+/+/−^ samples, but the difference did not reach the significant level compared to WT littermates. **g** The PGF2a/PGE2 ratio was not significantly different between WT, Ts1Cje and Ts1Cje;*Cbr1*^+/+/−^. Statistical significance was assessed using one-way ANOVA.

Cbr1 has been shown to convert prostaglandin E2 (PGE2) into prostaglandin F2a (PGF2a)^26,27^. We thus investigated whether amounts of PGE2 and PGF2a were altered by the overexpression of Cbr1 in the hippocampus of DS model mice (Fig. 7). Using MS/MS chromatography, we detected endogenous PGE2 and PGF2a, using internal standards (PGE2-d4 and PGF2a-d4) for precise quantification (Fig. 7a–d). Quantitative analysis revealed a significant decrease in PGE2 and PGF2a in the hippocampus of Ts1Cje mice compared to their WT littermates (Fig. 7e,f). There was a tendency for an increase in Ts1Cje;*Cbr1*^+/+/−^ samples even though this did not reach the significance level when compared to the Ts1Cje group. However, there was no statistical difference between Ts1Cje;*Cbr1*^+/+/−^ and WT mice, implicating partial recovery of PGE2 and PGF2a levels by resumption of *Cbr1*. On the other hand, the PGF2a/PGE2 ratio was not significantly different between Ts1Cje, Ts1Cje;*Cbr1*^+/+/−^ and WT groups (Fig. 7g). Overall, these results show that PGE2 and PGF2a are significantly reduced in the Ts1Cje brain and suggest that *Cbr1* copy number resumption rescues this phenotype partially. The lack of a change in the PGF2a/PGE2 ratio argues against an acceleration of the conversion of PGE2 into PGF2a, indicating that the decrease in PGE2 has a different origin.

### *Cbr1* resumption does not rescue the decrease in the depolarization envelope

Among the genes in the candidate region, *Cbr1* seemed to be one of the possible responsible genes for the regulation of GABA_B_ receptor-dependent inhibition of the later phase of the depolarization envelope since PGE2 stimulates the synthesis of estradiol^28^, one of neurosteroids that is demonstrated to attenuate GABA_B_ receptor-mediated responses in the brain^29^. Therefore, we compared the depolarization envelope between Ts1Cje and Ts1Cje;*Cbr1*^+*/*+*/*−^ littermate mice, and found that there was no statistical difference (*p* = 0.747, Student’s t-test) in the depolarization envelope at 950 ms between Ts1Cje (95.0 ± 6.4%, *n* = 45) and Ts1Cje;*Cbr1*^+*/*+*/*−^ (98.1 ± 7.3%, *n* = 29) mice, suggesting that the inhibition of the later phase of the depolarization envelope was not rescued in Ts1Cje;*Cbr1*^+*/*+*/*−^ mice.

## Discussion

In the present study, we found that the later phase of the depolarization envelope during LTP-inducing stimulation, which was regulated by GABA_B_ receptors (Fig. 2), was attenuated in Ts1Cje (Fig. 1a) and Ts2Cje (Fig. 1b) mice, but not in Ts1Rhr mice (Fig. 1c), compared to WT mice. Moreover, the difference of the later phase of the depolarization envelope between the genotypes was canceled by the GABA_B_-receptor antagonist CGP55845 (Fig. 3), indicating that the GABA_B_ receptor was involved in this phenotype. Furthermore, the inhibitory/excitatory (I/E) ratio, or the ratio of the amplitude of GABA_B_ receptor-mediated IPSCs to that of AMPA receptor-mediated EPSCs, was larger in Ts1Cje than that in WT mice, while the ratio in Ts1Rhr mice was not (Fig. 4). These data suggest that the gene(s) included in the trisomic region of Ts1Cje but not in that of Ts1Rhr is responsible for the increase in GABA_B_ receptor-dependent inhibition of the later phase of the depolarization envelope. We further tried to identify the responsible gene using *Cbr1*-resumed (Ts1Cje;*Cbr1*^+/+/−^) mice. Behavioral experiments suggested possible involvement of Cbr1 in hippocampus-dependent learning (Fig. 6), while the decreased later phase of the depolarization envelope (Fig. 1) and reduced amount of PGE2 (Fig. 7) observed in Ts1Cje mice were not rescued.

It is generally believed that GABA_B_ receptor-mediated synaptic responses are not directly reflected in field potentials, and the effect of the GABA_B_-receptor antagonist found in this study is indirectly mediated by other factors, presumably by the regulation of NMDA receptor-mediated EPSPs. Since LTP induced in the same condition as that in which the depolarization envelope was examined was indistinguishable between genotypes (Supplementary Fig. 2), we speculated that NMDA receptor-mediated synaptic responses were also indistinguishable. However, a previous paper^20^ reports that NMDA receptor-mediated synaptic responses are different in Ts65Dn mice, although they were analyzed in the dentate gyrus, not in the CA1 region in which the present study was conducted. Because the NMDA receptor-mediated synaptic responses seemed indistinguishable in the CA1 region, we considered that the observed difference in the effect of the GABA_B_-receptor antagonist on the depolarization envelope was caused by the difference of GABA_B_ receptor-mediated synaptic inhibition in this region between genotypes. To support this idea more directly, we examined isolated GABA_B_ receptor-mediated synaptic currents in the whole-cell recording (Fig. 4), and actually, we identified the significant difference in this measure.

GABA_B_ receptors inhibit neuronal excitability and modulate synaptic plasticity^30^. Ts65Dn mice show GABA_B_ receptor-dependent attenuation of LTP in the perirhinal cortex^31^ and dentate gyrus^20^, which suggests the involvement of GABA_B_ receptors in intellectual disability in DS patients. Although GABA_B_ receptor-mediated synaptic currents were increased, there was no attenuation of LTP in the CA1 region in our conditions (Supplementary Fig. 2). This finding would not necessarily be contradictory to the cognitive deficiency seen in Ts1Cje mice (Fig. 6), because the relationship between LTP and learning and memory is still controversial^32,33^. In contrast, abnormalities in LTP were found in Ts65Dn mice^12^, which were ameliorated by GABA_B_-receptor antagonists^20^. Electrophysiological experiments of those studies were performed in the dentate gyrus, while the present study was conducted in the CA1 region. Thus, the absence of the abnormality in LTP in this study may result from regional differences between the studies. Further study is required to identify the cause of this discrepancy.

Fundamental therapeutics for DS should require identification of responsible genes. Among the genes in the responsible region elucidated in our experiments, we wondered if the *Cbr1* gene was a key player of the GABA_B_ receptor-dependent decrease of the later phase of the depolarization envelope and the impairment in spatial memory accuracy. In rodents, the hippocampus is a well-known brain region in charge of spatial and working memory. Whereas there have been reports of impaired spatial learning and memory in Ts1Cje mice^6,34^, the conclusions regarding non-spatial working memory are controversial^12,18^. In the present study, we have focused on spatial learning and memory using the Barnes-maze task (Fig. 6). Although the learning performance of Ts1Cje mice appeared to be conserved (Fig. 6a–c), spatial memory accuracy was slightly worse than that in their WT littermates. The *Cbr1* resumption improved this deficiency (Fig. 6d–g), suggesting a role played by Cbr1 in hippocampus-dependent spatial memory.

We hypothesized that the increase in the *Cbr1* expression level in Ts1Cje mice would accelerate the degradation of PGE2 into PGF2a^26,27^. In the brain, PGE2 stimulates the production of estradiol, and can affect neuronal properties and activity^28^. Estradiol has notably been shown to strongly diminish the response to GABA, and more specifically that through GABA_B_ receptors^29,35^ . This led us to speculate that a decrease in PGE2 in the hippocampus of DS could play a role in the over-inhibition through aberrant regulation of estradiol in the central nervous system. We indeed found a significant decrease in PGE2 in the hippocampus of Ts1Cje mice (Fig. 7) along with signs of increased GABAergic inhibition onto CA1 pyramidal cells (Figs. 2, 3, 4), which could fit this hypothesis. However, the fact that the copy number resumption of *Cbr1* did not recover the GABA_B_ receptor-dependent over-inhibition indicates that *Cbr1* is unlikely involved in this abnormality. Moreover, if Cbr1 was indeed accelerating the degradation of PGE2 into PGF2a, an increase in PGF2a would be expected, which was not the case in the brain of Ts1Cje mice. On the contrary, we observed a significant decrease in PGF2a amount in hippocampal extracts. The decrease we observed in hippocampal PGE2 is thus likely to have a different origin from the mechanistic standpoint, and future work will help elucidate how this reduction in PGE2 occurs in the DS brain. Finally, the fact that the *Cbr1* resumption to two copies was able to recover the deficit in spatial memory accuracy in Ts1Cje mice without acting significantly on PGE2 quantity or GABAergic over-inhibition suggests that Cbr1 is associated with the memory impairment, but electrophysiological and biochemical phenotypes are independent of Cbr1 and caused by other gene(s) that is included in Ts1Cje but not in Ts1Rhr.

The mechanism and therapeutics of DS are yet to be unveiled. Our study and future investigation on responsible gene(s) using mouse models and the gene-resumption system should contribute to the clarification of DS pathology.

## Methods

### Housing of mice and conditions for behavioral experiments

All animal breeding and experimental procedures were performed in accordance with the guidelines of the Animal Experiment Committee of RIKEN Brain Science Institute and Fujita Health University and the Animal Care and Experimentation Committee of University of Tokyo. The experiments were approved by the Animal Experiment Committee of RIKEN Brain Science Institute and Fujita Health University and the Animal Care and Experimentation Committee of University of Tokyo. In this study, we used a minimum number of mice that were required to draw the conclusions and tried to minimize their suffering as much as possible. Adult male mice were used in all the experiments. Animals were maintained on a 12-h light/dark cycle with ad libitum access to food and water. Behavioral tests were conducted in the light phase of the light/dark cycle and mice were given a minimum of 30-min habituation to the experimental room before the start of the tests.

### Mouse lines

Ts(16C-tel)1Cje mice, referred to as Ts1Cje mice, were maintained by backcrossing Ts1Cje males with C57BL/6 J females for N16 generations^36^. *Cbr1* KO line was generated by in-vivo Cre/loxP recombination using a similar strategy to that previously described^17^ (Supplementary Fig. 3a). Briefly, a loxP site and a frt-flanked neomycin resistance cassette were inserted upstream from *Cbr1* exon 1 and a second loxP site was inserted downstream from *Cbr1* exon 2. This recombinant construct was inserted in genomic DNA by homologous recombination in embryonic stem cells, which were then injected into blastocysts to obtain a flox-*Cbr1* mouse line. Heterozygous flox-*Cbr1* females were then mated to Tg(EIIa-cre)C5379Lmgd males^37^, resulting in the excision of exons 1 and 2 of *Cbr1*. Effective deletion was confirmed by Sanger sequencing. The genotype of *Cbr1* KO mice was determined by PCR using the following primers: *Cbr1*-Fw: CAACAAACACACCCCCCACCA; *Cbr1*-Rev: GCTGGAAGACATGGCTGCGT and *Cbr1*-Ex3Rev: GTTCATGAGCCCCACCAGCT amplifying PCR products of 207 bp for the WT allele and of 430 bp for the mutant allele (Supplementary Fig. 3c).

### Slice preparation

Transverse hippocampal slices of 3–5-month-old WT, Ts1Cje, Ts2Cje, Ts1Rhr, Ts1Cje;*Cbr1*^+/+/−^ and C57BL/6 J mice were prepared as previously described^38^. Briefly, mice were anesthetized deeply with halothane or isoflurane and decapitated, and then the brains were removed. Transverse hippocampal slices (400-µm thick) were cut with a tissue slicer (Vibratome 3000, Lancer, St Louis, MO, USA or LEICA VT 1200S, Leica Biosystems, Nussloch, Germany) in the Krebs–Ringer external solution containing (in mM): 119 NaCl, 2.5 KCl, 1 NaH_2_PO_4_, 26.2 NaHCO_3_, 11 glucose, 2.5 CaCl_2_ and 1.3 MgSO_4_, which was saturated with 95% O_2_ and 5% CO_2_. The slices were incubated for at least 1 h at room temperature in the interface-type holding chamber filled with the external solution, and then a slice was transferred to the submersion-type recording chamber.

### Extracellular field-potential recordings

Synaptic responses were recorded using Axopatch 1D or MultiClamp 700A (Molecular Devices, Sunnyvale, CA, USA) amplifiers, and the signal was digitized with Digidata 1322A (Molecular Devices), analyzed with pClamp9 or pClamp10 (Molecular Devices) and stored on a personal computer^38^. Synaptic responses were recorded at 25.0 ± 0.5 °C with extracellular field-potential recordings in the stratum radiatum of the CA1 region. The external solution was the same as that used for cutting slices, which was perfused at 1.5–2.0 ml/min in the recording chamber. To evoke synaptic responses, Schaffer collateral/commissural fibers were stimulated at 0.1 Hz (200-µs duration) with a bipolar tungsten electrode. In some recordings, picrotoxin (Sigma-Aldrich Japan, Tokyo, Japan) or CGP55845 (Tocris Bioscience, Avonmouth, UK) was included in the external solution. The CA3 region was surgically cut off to prevent bursting activity. A bipolar tungsten stimulating electrode and a recording glass pipette (about 1 MΩ when filled with 3-M NaCl) were placed in the stratum radiatum to stimulate Schaffer collateral fibers and to record field EPSPs, respectively. The stimulus strength was adjusted to evoke EPSPs with the slope value of 0.10–0.15 mV/ms. After EPSPs stabilized for 30 min, LTP was induced by tetanic stimulation (100 Hz, 1 s), and then, EPSPs were recorded further for 1 h.

### Whole-cell patch-clamp recordings

Whole-cell patch-clamp recordings were made from pyramidal cells in the CA1 region^38^. A recording patch pipette (3–6 MΩ) was filled with the internal solution containing (in mM): 122.5 K-gluconate, 17.5 KCl, 10.0 HEPES, 0.2 EGTA, 2.0 MgATP, 0.3 Na_3_GTP and 8.0 NaCl (pH 7.2, 295–305 mOsm). The series resistance and the input resistance were monitored throughout the experiment, and if they changed by more than 20% or the series resistance was above 30 MΩ, the data were discarded. A bipolar tungsten stimulating electrode was placed in the same layer as in the field-potential recording. Cells were held at − 60 mV (the liquid-junction potential was not corrected) and AMPA receptor-mediated EPSCs were recorded, which were adjusted to be 200–300 pA in amplitude. When GABA_B_ receptor-dependent IPSCs were recorded, picrotoxin (100 µM) was added to the external solution. After recording stable EPSCs for 10 min, non-N-methyl-d-aspartate (NMDA)-receptor antagonist CNQX (10 µM) and the NMDA-receptor antagonist d-(−)-2-amino-5-phosphonopentanoic acid (50 µM) were added, and the GABA_B_ receptor-mediated IPSC was recorded for further 10 min. The amplitude of GABA_B_ receptor-mediated IPSCs was measured by averaging the value for 10 ms around the peak. In some experiments, we confirmed that the isolated IPSCs were blocked completely by CGP55845 (not shown).

### Analysis for electrophysiology

The maximal initial slope of EPSPs was calculated in LTP experiments. The slopes of 6 consecutive field EPSPs, corresponding to 1 min, were averaged, and normalized to the mean slope of the 30-min baseline. The normalized average from 50 to 60 min after tetanic stimulation was compared by Student’s t-test between the genotypes. In the analysis of the depolarization envelope during the LTP-inducing tetanus, the amplitude of the depolarization envelope at 50 ms and 950 ms after the first stimulus of the tetanic stimulation was measured and normalized to the amplitude of the EPSP obtained by averaging baseline EPSPs for 30 min. Artifacts of the sample traces in field-potential recordings were truncated for clarity. The depolarization envelope (%) was compared between the genotypes or the conditions by Student’s t-test or one-way ANOVA with Bonferroni post-hoc test, respectively. The IPSC/EPSC (I/E) ratio was calculated by dividing the mean amplitude of the 10-min IPSC by that of the 5-min EPSC. The value of the I/E ratio was first corrected by Smirnov-Grubbs test and then compared between the genotypes by Student’s t-test.

### Gene-expression analysis by qRT-PCR

Brains were collected from adult mice under deep anesthesia and total RNA was extracted using Trizol reagent. cDNA synthesis was realized as previously described^36^ using the SuperScript II reverse transcriptase kit (Invitrogen, Tokyo, Japan). *Cbr1* and *Gapdh* (the housekeeper gene used for normalization) expression levels were analyzed using Taqman MGB probes and primers kits (Assays-on-Demand Gene Expression Products: Applied Biosystems, Tokyo, Japan). Quantitative measurement of cDNA levels was done in triplicates using an RG-3000 qPCR thermocycler (Corbett Research, Germantown, MD, USA) and they were analyzed using manufacturer’s software (Rotor-Gene Real-Time Analysis Software 6.0: Corbett Research).

### General health and neurological screening

At 21 weeks, body weight, body temperature and neuromuscular strength were recorded. A grip-strength meter (O’HARA & Co., Tokyo, Japan) was used to assess forelimb grip strength. Mice were lifted by holding the tail, allowing them to grasp a wire grid. They were then gently pulled until they released the grid and the maximum force applied by the forelimbs was recorded. Each mouse was tested three times and the highest value was used for data analysis. In the wire-hang test, mice were placed on a wire mesh, which was then slowly inverted, requiring animals to grip the wire in order not to fall off. Latency to fall was recorded, with 60-s cut-off time.

### Open-field test

Mice, aged 22 weeks, were placed in a 40 × 40 cm square open field homogeneously illuminated at 100 lx and allowed to freely explore for 120 min. Data on the travelled distance, moving speed and location of the mouse were acquired and analyzed automatically using manufacturer’s tracking software (Accuscan Instruments, Columbus, OH, USA). Laser sensors were placed on the walls of the open field, which were set a few centimeters above the floor but higher than the back of the mouse. When the mouse stood up on its hind legs, it would cross the laser beams, the number of which was counted as rearing. Another set of laser sensors was placed even closer to the ground of the open field. When the mouse was moving around and cut across some of the laser beams repetitively, the sensors detected it as a repetitive movement, which was defined as a stereotypic event in the analysis.

### Barnes-maze test

The Barnes-maze^25^ apparatus consisted of a white circular board (100-cm diameter) with twelve holes evenly spaced at the periphery and large visual cues placed around the apparatus to be used as spatial landmarks. The board was brightly illuminated at more than 800 lx and elevated 75 cm above the floor. Mice, aged 23 weeks at the beginning of the test, were placed in a box at the center of the board and automatically released at the start of each session. Mice were first given 5-min habituation to the board and 30-s habituation to a black Plexiglas escape box. During the acquisition test, animals were subjected to a daily trial in which they were given a maximum of 5 min to explore the board, find and enter the escape box placed under one of the peripheral holes. The location of the box was randomized across the animals but remained constant for a given mouse. Probe tests (3-min free exploration without an escape box) were conducted 1 day and 1 month after the last training session. Data were acquired and analyzed using ImageBM software, which was developed by Tsuyoshi Miyakawa based on ImageJ program (National Institute of Health, Bethesda, Maryland, USA).

### Prostaglandin dosage in the hippocampus

The extraction of prostaglandins was performed according to the method described in the previous paper^39^. Briefly, brains were removed from the skull under deep anesthesia and the hippocampus was dissected on ice. The hippocampus was weighed and transferred to ice-cold methanol containing prostaglandin E2 and F2a standards (ratio 7:1:1, PGE2-d4 #314010 and PGF2a-d4 #316010: Cayman Chemical, Ann Arbor, MI, USA). Samples were homogenized by sonication and vortexing and centrifuged for 15 min at 10,000 g at 4 °C. Supernatants were then sampled, incubated for 30 min at − 80 °C and centrifuged at 1,000 g for 10 min. Supernatants were finally sampled and used for quantitative analysis by MS/MS liquid chromatography.

Samples were separated on EASY-nLC 1000 using NANOHPLC capillary column C18 (Nikkyo Technos, Tokyo, Japan) with a trap column and analyzed by triple-quadrupole MS (TSQ Vantage EMR: Thermo Fisher Scientific, Tokyo, Japan) in the Selected Reaction Monitoring (SRM) mode. Data were processed using PinPoint software (Thermo Fisher Scientific) for peak area calculation, using internal controls PGE2-d4 and PGF2a-d4 for normalization.

### Statistical analysis

All data are presented as mean ± standard error of the mean (SEM). “*N*” represents the number of mice and “*n*” represents the number of samples or slices. When asterisks are not shown in the figures, it indicates that there is no statistical difference. Statistical significance is shown as *p < 0.05; **p < 0.01; ***p < 0.001. Student’s t-test, one-way ANOVA or two-way ANOVA was used for statistical analysis, except where otherwise stated.

## Supplementary information

Supplementary file 1

Supplementary file 2

Supplementary file 3

Supplementary file 4
