# Supplementary material for: Impairment of spatial memory accuracy improved by Cbr1 copy number resumption and GABAB receptor-dependent enhancement of synaptic inhibition in Down syndrome model mice
Source: Sci Rep. 2020 Aug 25;10:14187. doi: 10.1038/s41598-020-71085-9 (PMC7447763; doi:10.1038/s41598-020-71085-9)
Supplement: Supplementary file 1 [file 41598_2020_71085_MOESM1_ESM.docx]

**Supplementary Information**

**Impairment of spatial memory accuracy improved by *Cbr1* copy number resumption and GABA_B_ receptor-dependent enhancement of synaptic inhibition in Down syndrome model mice**

Fumiko Arima-Yoshida^1#^, Matthieu Raveau^2#^, Atsushi Shimohata^2#^, Kenji Amano^2^, Akihiro Fukushima^1^, Masashi Watanave^1^, Shizuka Kobayashi^1^, Satoko Hattori^3^, Masaya Usui^4^, Haruhiko Sago^5^, Nobuko Mataga^4^, Tsuyoshi Miyakawa^3^, Kazuhiro Yamakawa^2,6*^ & Toshiya Manabe^1*^

^1^Division of Neuronal Network, Institute of Medical Science, University of Tokyo, Tokyo 108-8639, Japan. ^2^Laboratory for Neurogenetics, RIKEN Center for Brain Science, Wako-shi, Saitama 351-0198, Japan. ^3^Division of Systems Medical Science, Institute for Comprehensive Medical Science, Fujita Health University, Toyoake-shi, Aichi 470-1192, Japan. ^4^Research Resources Division, RIKEN Center for Brain Science, Wako-shi, Saitama 351-0198, Japan. ^5^Center for Maternal-Fetal, Neonatal and Reproductive Medicine, National Center for Child Health and Development, Tokyo 157-8535, Japan. ^6^Department of Neurodevelopmental Disorder Genetics, Institute of Brain Sciences, Nagoya City University Graduate School of Medical Sciences,

Nagoya-shi, Aichi 467-8601, Japan.

*Corresponding authors

email: yamakawa@med.nagoya-cu.ac.jp (K.Y.) or tmanabe-tky@umin.ac.jp (T.Manabe)

#Equally contributed to this study

**Supplementary Figure 1.** The trisomic regions of MMU16 in the Down syndrome mouse models. The Ts2Cje (Ts65Dn) trisomic region includes 94 orthologues, that of Ts1Cje mice includes 67 genes and that of Ts1Rhr mice includes 33 genes.

**Supplementary Figure 2.** There was no statistical difference in LTP in the DS mouse models compared with WT mice. **a** LTP in WT (151 ± 4.1%, *n* = 23) and Ts1Cje (145.1 ± 3.0%, *n* = 24, *p* = 0.199) mice. **b** LTP in WT (150.7 ± 5.9%, *n* = 12) and Ts2Cje (139.7 ± 6.1%, *n* = 12, *p* = 0.214) mice. **c** LTP in WT (146.5 ± 3.4%, *n* = 23) and Ts1Rhr (149.5 ± 2.9%, *n* = 21, *p* = 0.499) mice. Tetanic stimulation was applied at time 0. Inset: Sample traces of EPSPs in WT (left) and mouse models (right) recorded at the times indicated in the graph. Statistical significance was assessed using Student’s t-test.

**Supplementary Figure 3.** Creation of a *Cbr1* KO mouse line. **a** A loxP site was inserted together with a frt-flanked neomycin resistance cassette upstream from *Cbr1* exon 1 and a second loxP site was inserted downstream from exon 2 to generate a *Cbr1*-flox construct. Action of a Cre recombinase led to the elimination of exons 1 and 2 *in-vivo* producing a *Cbr1* KO allele. **b** This *Cbr1* KO mouse was used in a subtractive approach in combination with the Ts1Cje model of DS that carries an extra copy of the distal part of chromosome 16 (MMU16), bringing *Cbr1* back to two copies in compound Ts1Cje;*Cbr1*^+/+/-^ mice. **c** Three specific primers (*Crb1*-Fw, *Cbr1*-Rev and *Cbr1*-Ex3-Rev displayed in **a**) allowed the discrimination between a WT allele (207 bp: amplified by primers *Crb1*-Fw and *Cbr1*-Rev) and the mutant *Cbr1*-KO allele (430 bp: amplified by primers *Crb1*-Fw and *Cbr1*-Ex3-Rev). **d** The expression level of *Cbr1* assessed by quantitative RT-PCR showed a significant increase in Ts1Cje brain extracts and a significant decrease in *Cbr1*^+/-^ mice. The compound Ts1Cje;*Cbr1*^+/+/-^ brought back *Cbr1* expression to a level comparable to WT. Statistical significance was assessed using one-way ANOVA.
